# The Morphology of Simulated Trade‐Wind Convection and Cold Pools Under Wind Shear

**DOI:** 10.1029/2021JD035148

**Published:** 2021-10-13

**Authors:** K. C. Helfer, L. Nuijens

**Affiliations:** ^1^ Department of Geoscience and Remote Sensing Delft University of Technology Delft The Netherlands

**Keywords:** shallow convection, wind shear, congestus, trade wind, large‐eddy‐simulation, precipitation

## Abstract

A growing body of literature investigates convective organization, but few studies to date have sought to investigate how wind shear plays a role in the spatial organization of shallow (trade‐wind) convection. The present study hence investigates the morphology of precipitating marine cumulus convection using large‐eddy‐simulation experiments with zonal forward and backward shear and without shear. One set of simulations includes evaporation of precipitation, promoting cold‐pool development, and another set inhibits the evaporation of precipitation and thus cold‐pool formation. Without (or with only weak) subcloud‐layer shear, conditions are unfavorable for convective deepening, as clouds remain stationary relative to their subcloud‐layer roots so that precipitative downdrafts interfere with emerging updrafts. Under subcloud‐layer forward shear (FS), where the wind strengthens with height (a condition that is commonly found in the trades), clouds move at greater speed than their roots and precipitation falls downwind away from emerging updrafts. FS in the subcloud layer appears to promote the development of stronger subcloud circulations, with greater divergence in the cold‐pool area downwind of the original cell and larger convergence and stronger uplift at the gust front boundary. As clouds shear forward, a larger fraction of precipitation falls outside of clouds, leading to more moistening within the cold pool (gust front).

## Key Points

- Without cold pools, new convection is triggered upwind of existing cells, and with cold pools, new convection is triggered downwind
- In the absence of (or with only weak) subcloud‐layer shear, rain falls into emerging updrafts, hindering the convective development
- Uplift at the cold‐pool gust fronts is strongest under subcloud‐layer forward shear (increasing wind speed with height)

## 1 Introduction

Triggered by the World Climate Research Programme's grand challenge on clouds, circulation, and climate sensitivity (Bony et al., 2015), tremendous research efforts have been undertaken in recent years to study maritime shallow clouds, with an increasing interest in their organization. A culmination was the ${EUREC}^{4}$A field campaign in 2020 (Stevens et al., 2021), which also motivated the successful classification of trade‐wind cloud patterns by their visual appearance from space into classes called fish, flower, sugar, and gravel (Stevens et al., 2019). This classification indicates that the dominant pattern of trade‐wind convection is not the unorganized, nonprecipitating cumulus humilis cloud (sugar) but rather the somewhat deeper, precipitating congestus (gravel) that may have a stratiform outflow (flower) at greater heights (Schulz et al., 2021). This finding motivates us to shed more light specifically on cumulus congestus clouds from large‐eddy‐simulations (LESs) using a setup that differs from the traditional BOMEX and ATEX cases that have been intensely used in the past decades (Nuijens & Siebesma, 2019).

Surface wind speed (and to lesser extent wind shear) is considered as one of the predictors of the aforementioned cloud patterns (Bony et al., 2020; Schulz et al., 2021). Helfer et al. (2020) (hereafter: HNRS20) ran idealized LES to investigate the effect of wind shear on trade‐wind cumulus convection, differentiating between backward shear (BS), where surface winds weaken with height, and forward shear (FS), where surface winds strengthen with height. Indicative of their representativeness of the trades, these simulations are dominated by clouds that resemble gravel, which sometimes have stratiform outflows near cloud tops that resemble flowers. A main result in HNRS20 is that any absolute amount of wind shear limits the strength of cloud updrafts because of a stronger downward‐oriented pressure perturbation force (as found in studies of deep convection, e.g., Peters et al., 2019). As a consequence, cloud deepening is hampered in the presence of shear. However, under FS, convection appears to have a tendency to grow deeper, which seems related to this system's enhanced potential to aggregate column moisture on mesoscales. Another noteworthy observation of HNRS20 is that wind anomalies within cold pools depend on the direction of the shear. This may hint at a possible role of downdrafts introducing different cloud‐layer momentum in the surface and subcloud layers. In modeling studies of deep convective cold pools, convective momentum transport (CMT) has been found to significantly influence cold‐pool winds (Grant et al., 2020; Mahoney et al., 2009). HNRS20 speculated about the role of wind shear in the triggering of new convection at cold‐pool edges.

It has long been known that cold‐pool edges can trigger secondary convection (e.g., Intrieri et al., 1990; Warner et al., 1979; Weckwerth & Wakimoto, 1992; Zipser, 1969) for which several (not necessarily mutually exclusive) mechanisms are being discussed in the literature. A purely thermodynamic mechanism involves enhanced moisture and thus buoyancy at the edges of cold pools, favoring convection (Romps & Jeevanjee, 2016; Seifert & Heus, 2013; Tompkins, 2001). Using a cloud‐resolving model, Tompkins (2001) showed that during the development of deep convective cold pools, evaporation of precipitation cools and moistens the boundary layer. The cold pool's gust front is consequently moister than the cold‐pool center. The lowered temperature can quickly recover, which removes nearly all convective inhibition and allows new convection to develop in response to minimal lifting. In the reduced entrainment “near environment” hypothesis (Böing et al., 2012; Schlemmer & Hohenegger, 2014), the interplay of moisture aggregation at cold‐pool edges (as opposed to the depletion of moisture inside cold pools) and vertical uplift at the leading edge of the cold pool's gravity current promotes the formation of wider, and thus deeper clouds less affected by entrainment. Gaining ground in recent literature is the dynamical or mechanical mechanism, whereby the leading edge of the cold pool's spreading gravity current is associated with a band of horizontal convergence in the wind field, which triggers uplift (Böing et al., 2012; Li et al., 2014; Meyer & Haerter, 2020; Torri et al., 2015; Xue et al., 2008). As moist near‐surface air is lifted to higher levels above the level of free convection, it can moisten the upper boundary layer, lower the troposphere, and trigger new convective events. This forced uplift may be enhanced by the collision of two or more cold‐pool fronts (e.g., Feng et al., 2015; Meyer & Haerter, 2020).

In their LES study of a specific RICO day, Li et al. (2014) found little evidence that supports a thermodynamic mechanism for shallow convection. Inspired by studies on midlatitude squall lines (Rotunno et al., 1988; Weisman & Rotunno, 2004), they pointed out a possible role of wind shear in the tilting of updrafts and clouds, which decides whether precipitation can fall into preexisting cold pools and possibly strengthen them. In their simulations, the vorticity of the cold‐pool boundary is weaker than that of the ambient wind profile, and the updraft thus tilts away from the cold pool, gaining access to converged moisture at the cold‐pool boundary, which is advantageous for convective development. Hence, it seems plausible that this process could help explain the cloud‐top height (CTH) differences between FS and BS that were reported in HNRS20. A recent study by Mulholland et al. (2021) focusing on squall‐line deep convection also notes that forced uplift is larger under stronger subcloud‐layer shear as it helps larger mass fluxes and deeper clouds.

In our present study, we aim to address why cloud deepening may be inhibited more under BS than under FS and, in particular, if this is dependent on the presence or absence of cold pools as suggested in HNRS20. We describe the morphology of shallow convective systems under shear in idealized large‐domain LES with and without the evaporation of precipitation. By turning off evaporation, we limit the formation of cold pools and thus the organization of convection in arc‐shaped bands surrounding cold pools. We utilized a computational domain of 50×50 ${km}^{2}$, which is sufficiently large for cold‐pool organization (Seifert & Heus, 2013).

The remainder of this paper is structured as follows. In the following section, we shortly review the simulation setup as well as the additional simulations we ran for the present paper. We then present the results in a twofold manner. First, we discuss the effects of wind shear on cold pools and the triggering of new convection at their fronts. Second, we ask how clouds behave under wind shear before cold pools emerge, by analyzing simulations in which cold‐pool formation is suppressed. Finally, we discuss and summarize our findings in the concluding section.

## 2 Experimental Design

**Figure 1:**
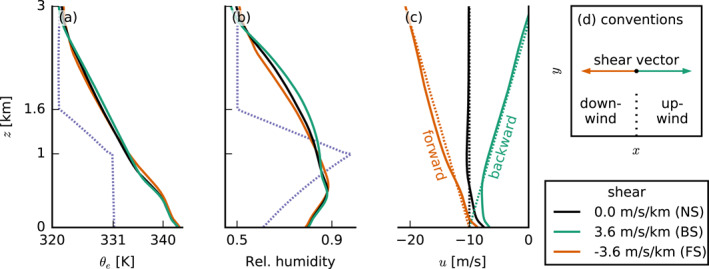
(a–c) Profiles of (a) equivalent potential temperature $\theta_{e}$, (b) relative humidity, and (c) the zonal wind components u. Dotted lines are initial profiles and solid lines indicate profiles that are averaged over the last 10 hr of the standard simulations. Orange stands for forward shear (FS), black for no shear (NS), green for backward shear (BS), and purple profiles are the same in all simulations. This color coding is the same for all other figures. (d) Schematic of the directional conventions used in this paper: downwind is in the negative x‐direction and upwind in the positive x‐direction.

We utilized the same experimental setup as in HNRS20 and only point out its most important aspects here. Using version 4.2 of the Dutch Atmospheric LES model (DALES; Heus et al., 2010), we simulated an idealized shallow cumulus case, typical of the North Atlantic trades (Figure 1). Our domain has a size of 50.4×50.4×17.9 ${km}^{3}$, with a grid spacing of 100 m in the horizontal and a nonuniform vertical grid (stretched from 10 m at the surface to 190 m at the top). Simulations were run for 48 hr to allow for the development of sufficient precipitation. Advection was computed by a fifth‐order scheme in the horizontal and a second‐order scheme in the vertical, and a Galilean transform was performed to reduce advective errors. We deployed a single‐moment microphysics scheme that includes ice and allows for precipitation (Böing et al., 2012; Grabowski, 1998). The model uses an isotropic eddy‐diffusivity approach to parametrize subgrid turbulence.

For the sensible and latent surface heat fluxes, we prescribed SHF=15.3 W $m^{-2}$ and LHF=225.2 W $m^{-2}$, respectively. These values allow for the development of the cloud species that we are interested in: cumulus congestus, which are somewhat deeper than shallow cumuli. The use of constant fluxes removes interactions between cold pools and surface fluxes, including those that could enhance or inhibit thermodynamic mechanisms of triggering convection. While over land interactive surface enthalpy fluxes are crucial for cold‐pool modeling, Gentine et al. (2016) suggested that over oceans they only matter for cold pools of scales much larger than our domain. The surface momentum flux was computed interactively by the model, which implies that simulations that develop stronger surface winds (e.g., under FS) also develop larger surface friction. Interactions between the density current and surface friction may matter for setting the scales of cold pools and organization (Stephan, 2021), but are not explored here. We applied a constant radiative cooling rate of −2.5 K/d to the liquid water potential temperature $\theta_{l}$. Large‐scale subsidence was calculated interactively, using a weak temperature‐gradient approach (Daleu et al., 2012). The total water specific humidity $q_{t}$ was nudged toward its initial profile above 4 km with a time scale of 6 hr to avoid spurious moisture tendencies.

To investigate the dependence of shallow convection and cold pools on vertical wind shear, we ran experiments with different wind profiles (Figure 1c). As discussed by HNRS20, BS, where surface easterlies weaken with height and turn westerlies eventually, is by far the most common in the North Atlantic trades. However, FS, where surface easterlies strengthen with height, occasionally occurs as well, in particular in July and August. The analysis of HNRS20 revealed distinct differences in the effect that shear has on convection when it is forward as opposed to backward. The authors further showed that the strength of shear does not play a major role. Hence, we here investigated three different zonal wind profiles with either NS (black line in Figure 1c), BS (green, $\partial_{z}u=3.6\times 10^{-3}$ $s^{-1}$), or FS (orange, $\partial_{z}u=-3.6\times 10^{-3}$ $s^{-1}$) (Note that our BS and FS cases correspond to the BS‐4X and FS‐4X cases of HNRS20, respectively). These wind profiles were used as both the initial profiles and the geostrophic forcing. We did not prescribe any meridional wind (v=0). In the calculation of the Coriolis acceleration, we take a latitude of 15° N.

**Figure 2:**
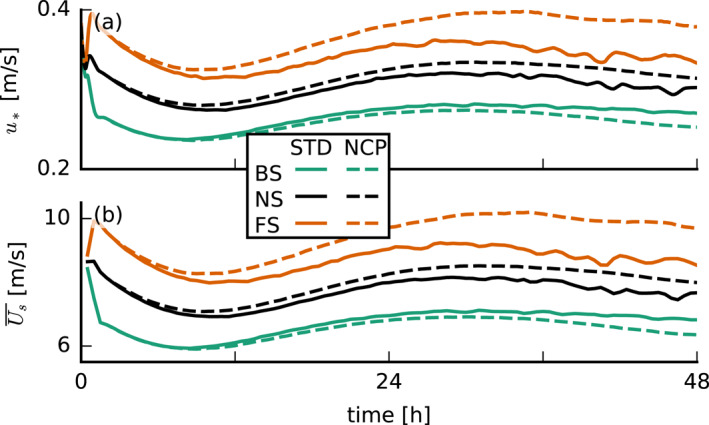
Time series of (a) the surface friction velocity $u_{*}$ and (b) the domain‐averaged total wind speed at 5 m height $U_{s}$. As explained in Figure 1, orange indicates forward shear (FS), black no shear (NS), and green backward shear (BS), while solid lines indicate the standard (STD) runs and dashed lines the no‐cold‐pool (NCP) runs. The line colors and types are the same in all the following figures, unless indicated otherwise.

It is important to realize that the wind profiles that develop during the course of the simulation differ from the initial profiles and the geostrophic forcing. After the initialization of the simulation, the winds evolve to reach an equilibrium after about 24 hr and stay approximately constant thereafter (Figure 2). Figure 1 shows the profiles from the end of the simulation with solid lines and the initial profiles with dotted lines. This reveals that in the subcloud layer, FS occurs even in the BS case, which is also a common feature of the trades (e.g., Holland & Rasmusson, 1973). The presence of FS in the subcloud layer is important throughout this paper.

In addition to one set of standard runs with each of the three wind profiles (labeled STD), we performed another set of experiments in which we suppressed the formation of cold pools (labeled NCP, no cold pools). To this end, we turned off the evaporation of precipitation in the LES, which Böing et al. (2012) showed to be very effective at suppressing cold pools. All precipitation in these simulations reaches the surface, and no latent cooling due to the evaporation of rain occurs, which is a crucial ingredient for the formation of cold pools (e.g., Khairoutdinov & Randall, 2006).

## 3 Cold Pools Under Shear

### 3.1 Cold‐Pool Structure and Behavior

**Figure 3:**
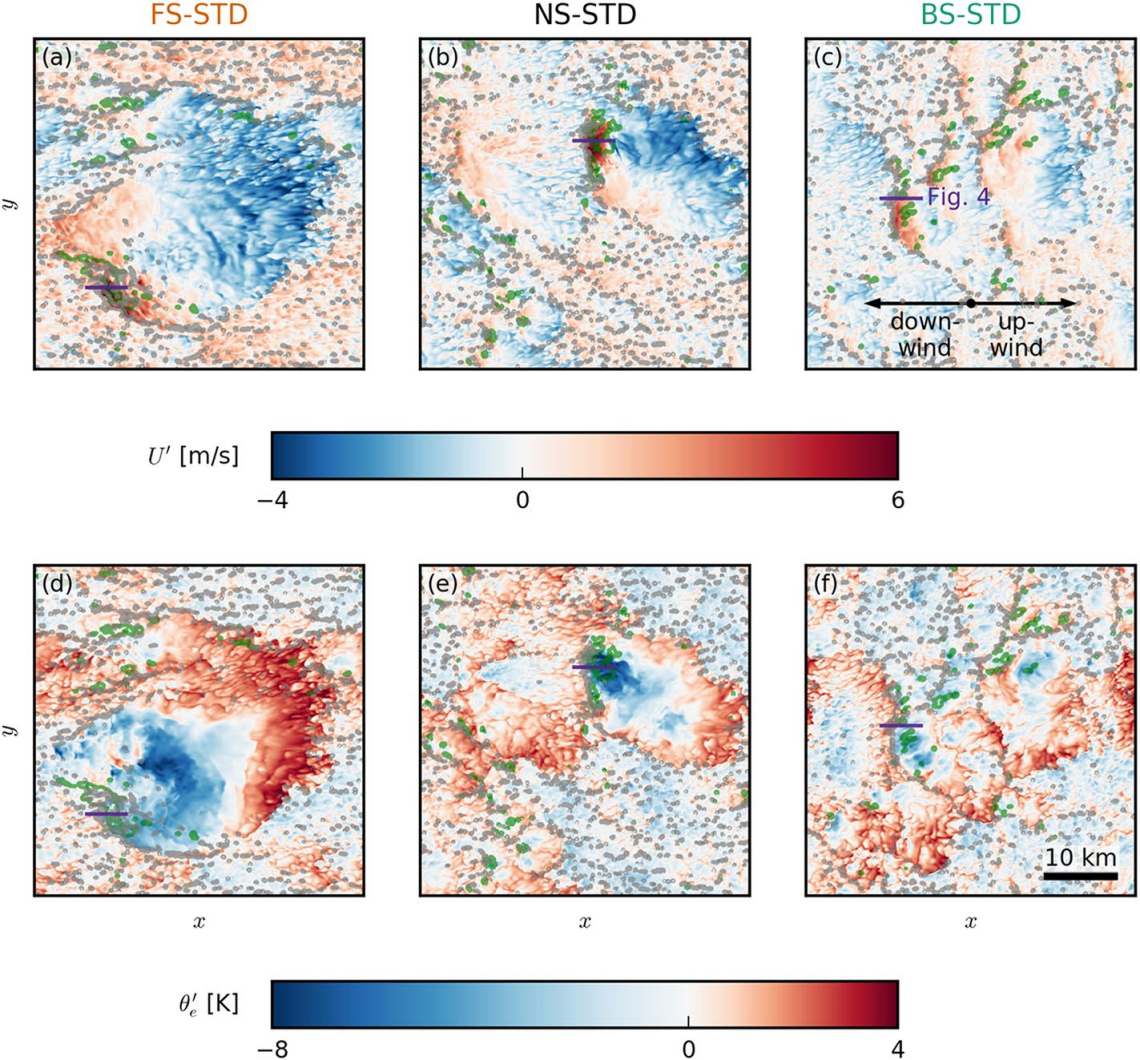
Snapshots of the large eddy simulation domains during exemplary cold‐pool events in the (a and d) forward shear (FS)‐standard (STD), (b and e) no shear (NS)‐STD, and (c and f) backward shear (BS)‐STD case. The colormaps in the x‐y cross section show (a–c) total wind speed deviations $U^{\prime }$ and (d–f) equivalent potential temperature deviations $\theta_{e}^{\prime }$ (both from the slab average) at the lowest model level (5 m). The gray outlines indicate strong updrafts in the subcloud layer (w=1 m/s at 400 m), and the green outlines indicate surface precipitation ($q_{r}>0$). The snapshots were taken around 40 hr. The cross sections of Figure 4 are marked in purple.

All our standard (STD) simulations are characterized by the gravel type of organization including cold pools (Figure 3). In Figure 3, we present top‐down views of the computational domain, showcasing the different structure of cold pools in our three shear cases. In these snapshots, the mean wind (∼u) blows from right to left (east to west), and hence, the left is referred to as downwind, the right as upwind (see also Figure 1d), and north would be at the top.

Cold‐pool formation starts with the precipitative downdraft (rain shaft) of a deep‐enough cloud. Near the surface, the cold and dense air mass spreads out laterally as a gravity current, which is reflected by the diverging wind patterns shown in Figures 3a–3c. In those snapshots, red areas have (total) wind speeds faster than the slab average and are most prominently found at the downwind front of the cold pool, where the gust front adds up to the mean wind speed. Conversely, on the upwind side of the cold pools, the cold‐pool front moves against the mean wind, leading to slower total wind speeds (shown in blue).

The cold pools have a characteristic thermodynamic signature (Figures 3d–3f). We make use of the equivalent potential temperature $\theta_{e}$, which combines information about the temperature T and the relative humidity H (Emanuel, 1994):

(1) $$\theta_{e}=T{\left(\frac{p_{0}}{p_{d}}\right)}^{R_{d}/\left(c_{pd}+c_{l}r_{t}\right)}H^{-R_{v}r_{v}/\left(c_{pd}+c_{l}r_{t}\right)}\exp\left(\frac{L_{v}r_{v}}{\left(c_{pd}+c_{l}r_{t}\right)T}\right),$$

where $p_{0}=1000$ hPa is a reference pressure; $p_{d}$ is the partial pressure of dry air; $R_{d}$ and $R_{v}$ are the gas constants for dry air and water vapor, respectively; $c_{pd}$ and $c_{l}$ are the heat capacities (at constant pressure) of dry air and liquid water, respectively; $r_{v}$ and $r_{t}$ are the mixing ratios of water vapor and total water, respectively, and $L_{v}$ is the latent heat of vaporization. Very low values of $\theta_{e}$ are found in the center of the cold pool, indicating that the air mass has its origin at higher altitudes where the air is cold and dry (see Figure 1). The outermost edges of the cold pool, especially on the upwind edge, have high values of equivalent potential temperature, which suggests convergence of moist air. Because the surface fluxes are held fixed, the spatial differences in temperature and humidity may be more persistent than in nature. While in the NS and FS cases, cold pools of significant size and strength occur (like the ones in Figures 3a and 3b), they are much smaller in the BS case (Figure 3c). As we will later elaborate, they also occur more rarely in the BS and the FS cases.

Similar to what observations show, our cold pools are usually not symmetric in their appearance. Visual inspection of a large number of scenes from our simulations shows that new convection (strong subcloud‐layer updrafts indicated in gray in Figure 3) is preferably triggered at the downwind edge of the cold pools (i.e., on the left in the panels of Figure 3), where strong winds and presumably large horizontal convergence lead to mechanical uplift (Mulholland et al., 2021).

**Figure 4:**
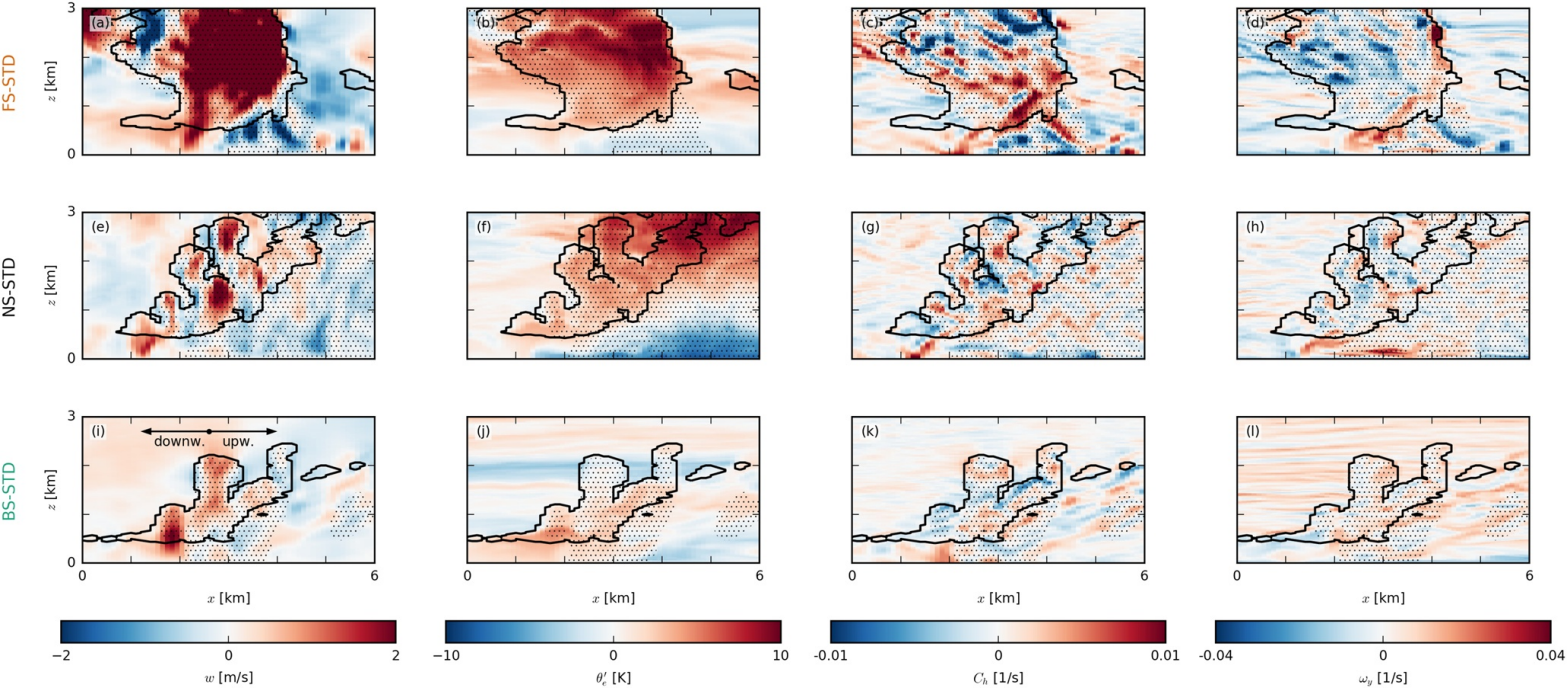
Snapshots of exemplary cold‐pool fronts in the (a–d) forward shear (FS)‐standard (STD), (e–h) no shear (NS)‐STD, and (i–l) backward shear (BS)‐STD cases. The colormaps in the x‐z slices show (first column) the vertical velocity w, (second column) the equivalent potential temperature anomaly $\theta_{e}^{\prime }$, (third column) the horizontal convergence $C_{h}$, and (fourth column) the meridional component of the vorticity $\omega_{y}$. In each panel, the black outlines indicate clouds (i.e., the $q_{l}=0$ isoline), and the dotted areas indicate precipitation. The location of each snapshot is marked in purple in Figure 3. Each panel is 6 km wide, averaged over 1 km in the meridional direction and taken from around 40 hr (the same times as Figure 3).

We further investigate the vertical cloud and boundary layer structure accompanying the exemplary cold pools from Figure 3 by presenting vertical x‐z cross sections (Figure 4). In each panel in Figure 4, a strong precipitative downdraft is located near the right edge of the excerpt, but note that in the FS and BS cases, precipitation is or has already ceased there (see Figures 4a, 4e and 4i). Focusing on the NS‐STD case (middle row), the cold pool itself is visible as a low‐temperature tongue (in terms of equivalent potential temperature $\theta_{e}$) extending from the right edge of the snapshot to nearly the x=1 km mark (Figure 4f). Ahead of this cold pool (downwind), updrafts and new clouds (secondary convection) are developing near cloud base (Figure 4e). Similar signatures of w and $\theta_{e}$ can be seen in the FS and BS cases. An important ingredient in the triggering of new convection by cold pools is the convergence that occurs at its downwind gust front (see Figures 3a–3c). Horizontal convergence, $C_{h}=-\partial_{x}u-\partial_{y}v$, between the front and the ambient wind is largest near x=1…2 km in Figures 4c, 4g, and 4k, where vertical uplift is also strong (Figures 4a, 4e, and 4i). In the FS and NS cases, there is also greater zonal wind shear in the density current (upwind tilting of the cold pool boundary) as reflected by positive values of the meridional vorticity, defined as: $\omega_{y}=\partial_{z}u-\partial_{x}w$ (Figures 4d, 4h, and 4l). In the mean or ambient wind, the subcloud‐layer vorticity is instead negative (left edge of Figures 4d, 4h, and 4l), as winds tend to increase with height away from the surface where they experience the strongest friction. In the FS and NS cases, the density current is apparently much stronger (compared to the BS case).

### 3.2 Convergence, Vorticity, and Uplift at Cold‐Pool Fronts

**Figure 5:**
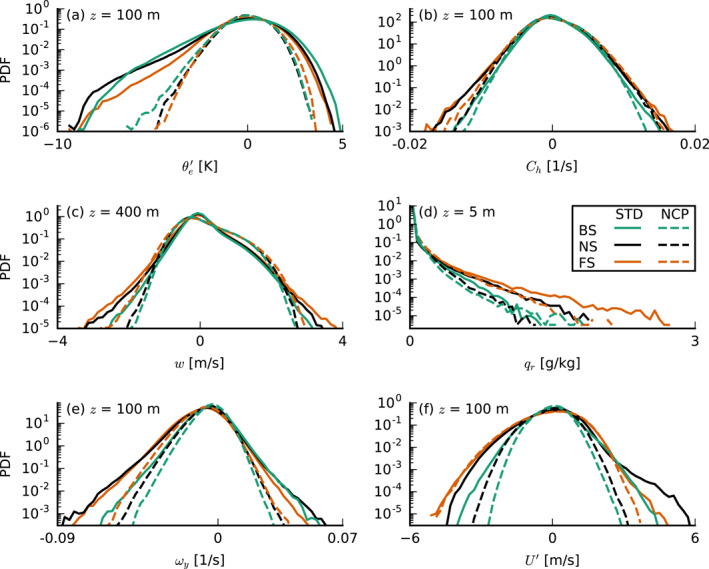
Probability density functions (PDFs) of (a) the equivalent potential temperature anomaly $\theta_{e}^{\prime }$ at 100 m, (b) the horizontal convergence $C_{h}$ at 100 m, (c) the vertical velocity w at 400 m, (d) the rainwater specific humidity $q_{r}$ at 5 m, (e) the meridional vorticity component $\omega_{y}$ at 100 m, and (f) the zonal wind velocity anomaly $u^{\prime }$ at 100 m, all averaged over 24–36 hr of each simulation. Solid lines indicate the standard (STD) simulations and dashed lines the no‐cold‐pools (NCP) simulations. BS, backward shear; FS, forward shear; and NS, no shear.

**Figure 6:**
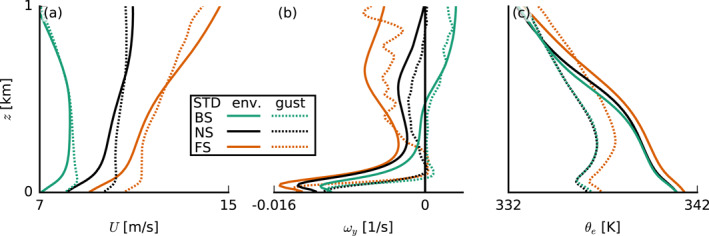
Composite profiles of (a) total wind speed U, (b) meridional vorticity $\omega_{y}$, and (c) equivalent potential temperature $\theta_{e}$ sampled over cold‐pool gust fronts ($\theta_{e}^{\prime }<-2$ K and $U^{\prime }>0$ at the lowest model level; dotted lines) and the environment ($\theta_{e}^{\prime }>-2$ K), all averaged over 24–36 hr of the standard (STD) simulations. BS, backward shear; FS, forward shear; and NS, no shear.

The above figures are merely some exemplary snapshots, but we may analyze probability density functions (PDFs) of the entire domain at specific heights to support these impressions (Figure 5). In addition, we construct composite profiles conditioned on all cold‐pool gust fronts as well as the ambient environment (Figure 6). To this end, we classify columns as belonging to a cold pool if $\theta_{e}^{\prime }<-2$ K at the lowest model level (where the prime indicates anomalies with respect to the slab average). The equivalent potential temperature is a commonly used quantity to identify cold pools (e.g., Schlemmer & Hohenegger, 2014; Zuidema et al., 2012). From this sample, we can identify the downwind gust front through positive anomalies of the total wind speed $U^{\prime }$ (see Figures 3a–3c). We focus on the period from 24 to 36 hr when convection is still shallow and cold‐pool fractions are small. Note that with our sampling approach it is not possible to capture profiles of convergence and updrafts at the gust front because they are located outside the cold pool (see Figure 4).

In the PDFs in Figure 5, we find indications of more vigorous cold‐pool gust fronts in the FS and NS cases. The figure shows a similar frequency of negative anomalies of $\theta_{e}$ in all STD cases (Figure 5a) but more frequent large values of horizontal convergence and divergence in the FS and NS cases (Figure 5b). These can be attributed to larger wind‐speed anomalies (Figure 5f). The FS and NS cases also have stronger subcloud‐layer updrafts (Figure 5c), which is in line with a more idealized study of deep convective cold pools by Mulholland et al. (2021) who showed that low‐level (forward) shear, which is pronounced in our FS and NS cases, leads to stronger, deeper, and wider squall‐line updrafts as well as an increased mass flux.

**Figure 7:**
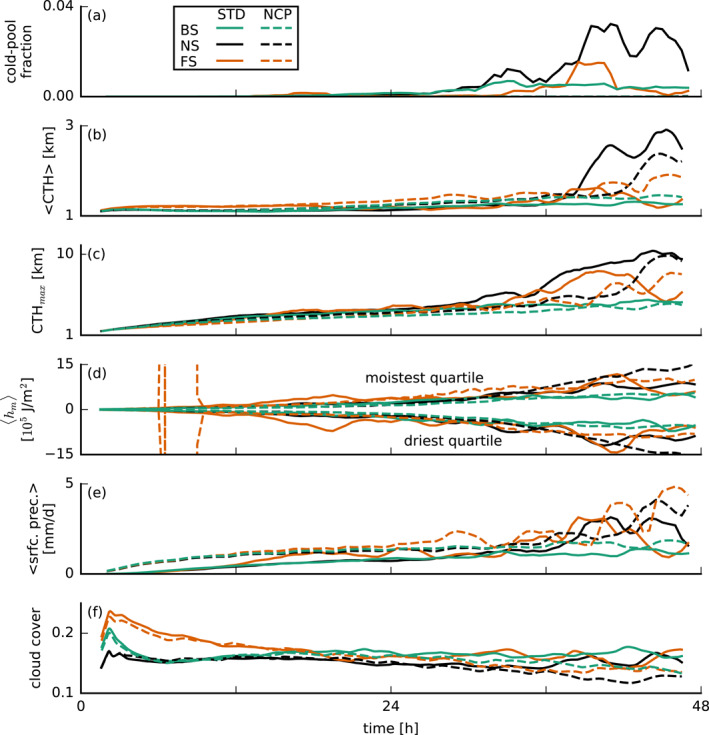
Time series of (a) the area fraction of cold pools ($\theta_{e}^{\prime }<-2$ K) at the lowest model level, (b) average and (c) maximum cloud‐top height (CTH), (d) vertically integrated (up to 1 km) moist static energy anomalies $<h_{m}>$ in the moistest and driest quartiles of 12.6×12.6 ${km}^{2}$ blocks, (e) surface precipitation, and (f) cloud cover. These data are smoothed using a 3‐hr running‐average filter. BS, backward shear; FS, forward shear; and NS, no shear.

Li et al. (2014) pointed out that the vorticity contrast between the cold‐pool front and the ambient wind profile sets the tilt of forced updrafts and therefore the degree to which they may tap into existing moist air in the cold pool front or in already moistened cloud air above the mixed layer and near cloud base (see their Figure 15). With a more pronounced negative vorticity in the ambient wind (Figure 5e), the updrafts are slanted forward more in the FS and NS cases than in the BS case, where the gust front has zero vorticity over a much deeper layer (Figure 6b). It is therefore unclear how a vorticity argument alone (as in the original RKW theory; Rotunno et al., 1988; Thorpe et al., 1982; Weisman & Rotunno, 2004) would lead to stronger updrafts in the FS and NS cases, because slanted updrafts are generally subjected to a stronger downward‐oriented pressure gradient force than updrafts that are upright. The FS case has a higher equivalent potential temperature in both the environment and the gust front (Figure 6c), due to larger absolute humidity (not shown), which may result from more evaporated precipitation during 12–24 hr of the simulation (see Figure 7e); as in the FS case, a larger fraction of rain falls outside of clouds (discussed in Section 4). The extra humidity would aid cloud development, but one can also imagine such differences to be quickly diminished in the presence of surface‐flux feedbacks (absent in our simulations).

The largest difference in the cold‐pool structure among our shear cases appears to be in the near‐surface wind speed. Figure 5f shows that the FS case, followed by the NS case, has larger negative and positive wind‐speed anomalies. This is not only true for the STD runs with cold pools, but also in the NCP runs where no gust fronts develop. Along with the stronger updrafts and downdrafts (Figure 5c), this implies that the FS case has stronger circulations (see also HNRS20). CMT might play a role here. In the presence of shear, vertical (convective) transport of momentum can introduce larger wind‐speed anomalies. Under FS, updrafts will carry slow surface winds, introducing convergence in a narrow updraft region through the depth of the mixed layer, while downdrafts (which are displaced downwind from the updrafts under FS, as discussed below in Section 4) introduce faster winds and broad regions of divergence in the raining areas. The downward transport of larger momentum may be even more pronounced in the presence of rain evaporation, as suggested in studies of deep convection (Grant et al., 2020; Mahoney et al., 2009). CMT can help sustain or even strengthen the cold‐pool circulations under FS. Instead, under BS the updrafts and downdrafts are not separated in space (Section 4), and the wind‐speed anomalies introduced by transport are weaker.

Because our simulations were run with constant and homogenous surface fluxes, differences in forced uplift we observe (Figure 5c) are not caused by thermodynamic fluxes, for example, the mechanism proposed by Tompkins (2001). The only difference being wind shear, it thus appears likely that the underlying cause of stronger uplift in the FS and NS cases (as compared to BS) lies in the process of momentum transport.

As discussed in HNRS20, moisture aggregation and precipitation in our simulations differ between the shear cases. In the time series in Figure 7, we show the cold‐pool fraction, defined as the area fraction where $\theta_{e}^{\prime }<-2$ K on the lowest model level; the average and maximum CTH; and deviations of moist static energy from the domain mean within the moistest and driest quartiles (in terms of total water path) of blocks of 12.6×12.6 ${km}^{2}$ compared to the domain mean (as a measure for moisture aggregations; see Bretherton & Blossey, 2017); the domain‐mean surface precipitation and the cloud cover. Even on the first simulation day, around 16 hr, the FS case begins to aggregate moisture (Figure 7d) and develop deeper clouds (Figures 7b and 7c), which rain more (Figure 7e) and form cold pools (Figure 7a). This sooner development of the FS case underlines that subcloud‐layer FS seems to favor stronger circulations, more divergence in the cold pool and more convergence and forced uplift at the outflow boundary.

Instead, the BS case seems to be at a disadvantage in the sense that it develops no deep clouds and significantly less cold pools (Figures 7a–7c). In the following section, we wish to shed more light on this and look more closely at the triggering of convection in simulations in which cold pools are suppressed (NCP).

## 4 Sheared Convection Without Cold Pools

### 4.1 System Development Without Evaporation of Precipitation

**Figure 8:**
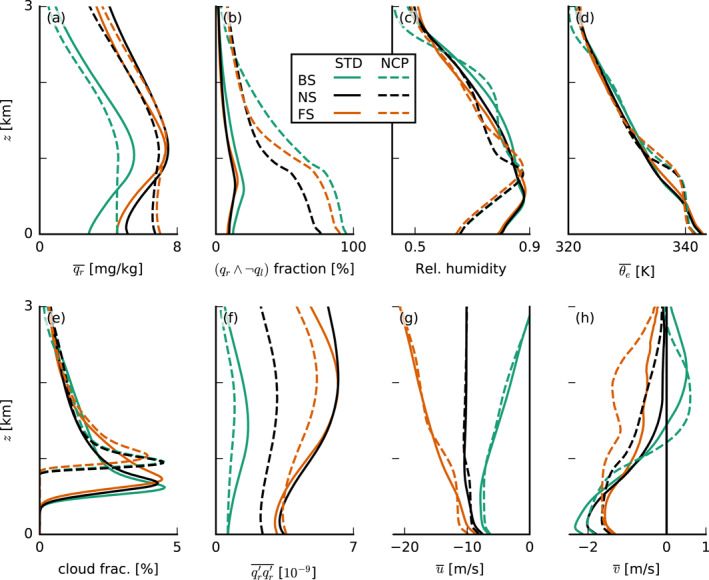
Slab‐averaged profiles of (a) rainwater specific humidity $q_{r}$, (b) the ratio of rainy grid points outside of clouds, (c) relative humidity, (d) equivalent potential temperature $\theta_{e}$, (e) cloud fraction, (f) the variance of $q_{r}$, (g) zonal wind velocity u, and (h) meridional wind velocity v, all averaged over the last 10  hr of each simulation. BS, backward shear; FS, forward shear; NCP, no‐cold‐pools; NS, no shear; and STD, standard.

Turning off the evaporation of precipitation (NCP runs) effectively suppresses cold pools (Figure 7a), but moisture aggregation is still a common feature (Figure 7d). Without cold pools, the thermodynamic structure of the simulated atmosphere is significantly different (Figure 8). While the amount of rain in the cloud layer differs only little (Figure 8a), surface precipitation is higher in the NCP runs than in the STD runs (see also Figure 7e) because in the NCP runs all the rain reaches the surface, while in the STD runs, a large fraction evaporates in the subcloud layer (Figure 8a). Consequently, in the NCP runs, more grid points outside of clouds contain rain compared to the STD runs (Figure 8b), while within clouds, the ratio is unchanged (not shown). The lack of rain evaporation in the subcloud layer leads to a decreased relative humidity there (Figure 8c). This is caused by both the lack of transfer of rain water to water vapor and by the lack of evaporative cooling, which results in a warmer subcloud layer (Figure 8d). Furthermore, we observe a higher cloud‐base height (Figure 8e) and a deeper mixed layer, for example, evident in the temperature, relative‐humidity, and zonal wind profiles (Figures 8c, 8d, and 8g), which contributes to the drier boundary layer. Without the evaporation of precipitation and thus cold pools, cloud tops are not significantly lower, but convective deepening is delayed to some extent (Figures 7b and 7c).

### 4.2 Convective Structure Along the Shear Vector

**Figure 9:**
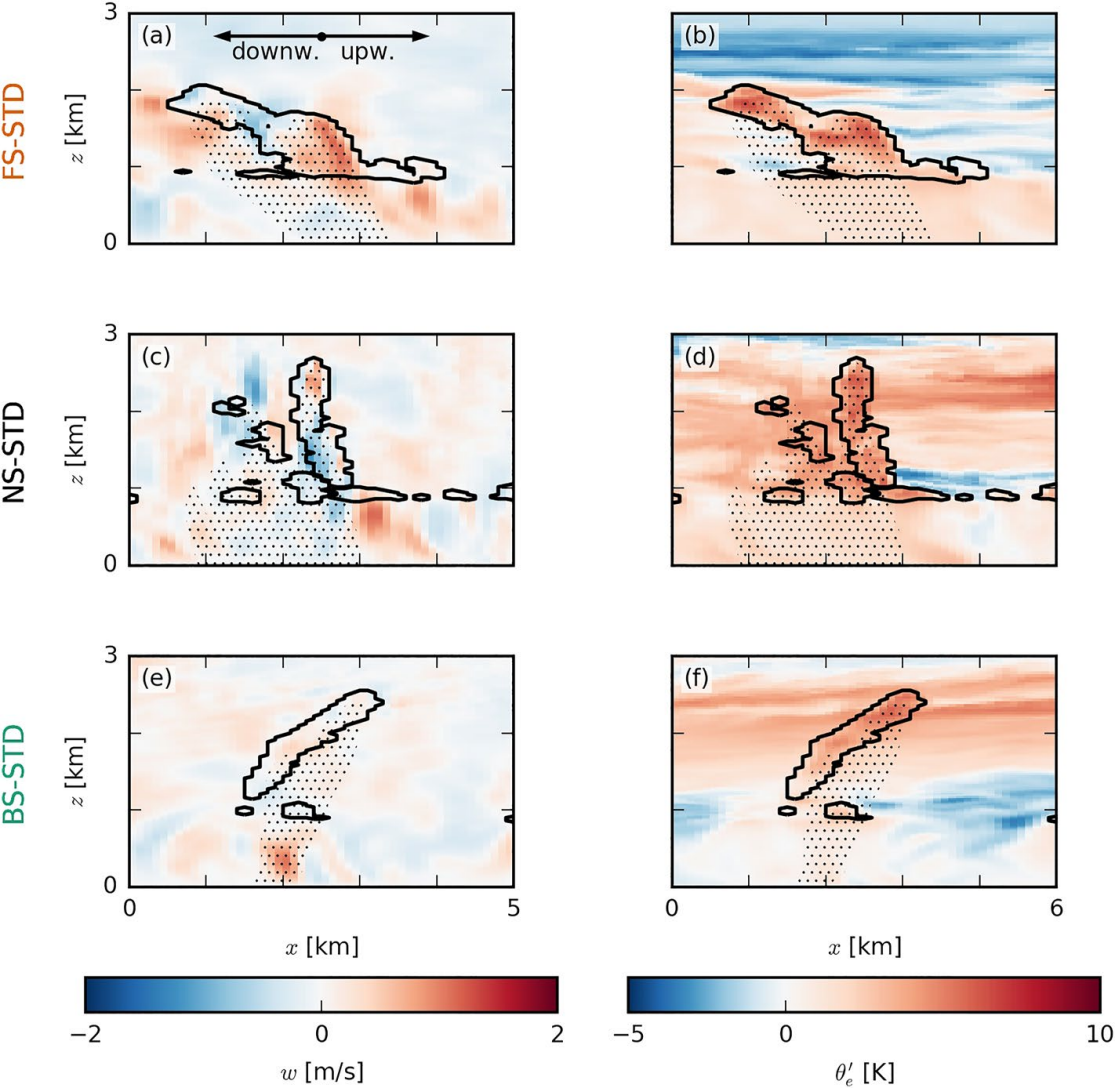
Snapshots of exemplary clouds in the (a and b) forward shear (FS)‐no‐cold‐pools (NCP), (c and d) no shear (NS)‐NCP, and (e and f) backward shear (BS)‐NCP cases. The colormaps in the x‐z slices show (left column) the vertical velocity w and (right column) the equivalent potential temperature anomaly $\theta_{e}$. Just as Figure 4, the black outlines indicate clouds (i.e., the $q_{l}=0$ isoline), and the dotted areas indicate precipitation. Each panel is 5 km wide, averaged over 1 km in the meridional direction and taken from the late stages of the simulation (around 40 hr) to allow for a comparison with Figure 4. STD, standard.

Exemplary snapshots of cloud systems from the NCP simulations (Figure 9) suggest that under FS and NS, precipitation is falling downwind from the clouds and downwind from the subcloud‐layer roots of the clouds, where new updrafts develop. Under BS, precipitation tends to fall near the existing subcloud‐layer updraft, which would essentially inhibit the updraft.

**Figure 10:**
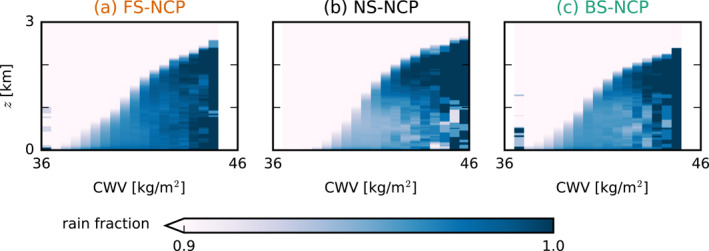
Composite profiles of the fraction of rainy grid points ($q_{r}>0$) averaged over bins of column‐integrated water vapor (CWV). All data are averaged from 30‐min output of the instantaneous 3D fields in the 12–18 hr of the no‐cold‐pools (NCP) simulations.

We may attempt to quantify where in our shear cases rain shafts are located in relation to the bulk of the clouds and liquid water. To this end, we organize the domain by column‐integrated water vapor (CWV), where high CWV corresponds to regions where moisture converges to form (deep) clouds. In some sense, mapping all grid points by CWV allows us to create a cross section through the bulk water vapor and cloud structure, moving from clear sky regions (low CWV) to cloud centers (high CWV). Figure 10 shows the distribution of precipitation as a function of height and CWV. The shear cases have somewhat different distributions of CWV, but nonetheless, differences in the distribution of rain are visible. Under NS and even more under FS, the presence of rain in columns with lower CWV is evident, whereas under BS, rain water below clouds is limited to the columns with highest CWV.

**Figure 11:**
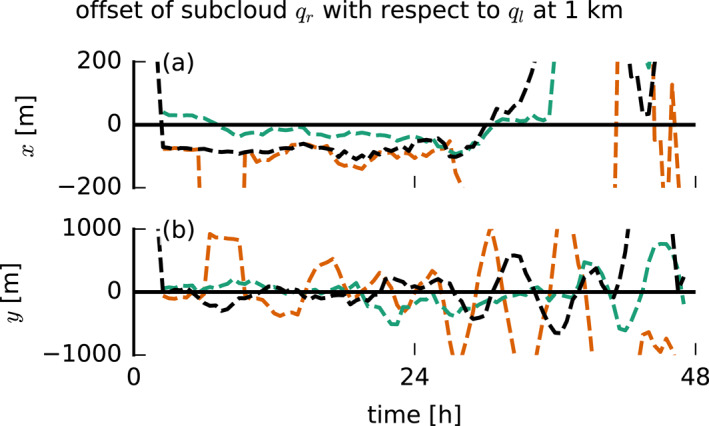
Lateral offset in (a) x and (b) y of the rainwater specific humidity field averaged over 0–1 km with respect to the liquid water specific humidity field at 1 km. The offset is computed from the center of mass of the matrix that contains the sum of the cross‐correlation vectors of each row of the $q_{l}$ field with every other row of the $q_{r}$ field (Equation 3). The analysis is done on a 30‐min output of the instantaneous 3D fields. For clarity, we only show the no‐cold‐pools simulations here.

The differences in the CWV‐binned cloud and rain distributions do not reveal whether rain is located upwind or downwind of clouds. To quantify the precipitation's preferred direction with respect to the clouds, we perform an analysis of the cross‐correlation of the cloud water field with the rainwater field. The cross‐correlation is a measure for the similarity of two vectors as a function of shift relative to each other, which is commonly used in signal processing. Occasionally, it is also used in atmospheric science, for example, to study coherent structures in the boundary layer (Lohou et al., 2000; Schmidt & Schumann, 1989). Generally, the cross‐correlation of two discrete real functions f and g of length N is defined by:

(2) $$X\left(\Delta \right)=\sum_{j=0}^{N}f\left(j\right)g\left(j+\Delta \right),$$

where Δ indicates the displacement (lag) of g with respect to f. We compute the cross‐correlation of every row i of the $q_{l}$ field (at 1 km, i.e., near cloud base) with every other row of the $q_{r}$ field (averaged over the subcloud layer up to 1 km) and sum up the resulting vectors. Making use of the periodicity of the fields (i.e., $N+\;i\;\hat{=}\;i$), this yields a matrix,

(3) $$X\left(\Delta_{i},\Delta_{j}\right)=\sum_{i=0}^{N_{i}}\sum_{j=0}^{N_{j}}q_{l}\left(i,\;j\right)q_{r}\left(i+\Delta_{i},j+\Delta_{j}\right),$$

with positive values where similarities between the two fields occur. The “coordinates” $\left(\Delta_{i},\Delta_{j}\right)$ of the center of mass of this matrix are assumed to form a good measure of the offset of the precipitation field with respect to the cloud field. The time series of these coordinates in Figure 11 shows a clear signal in the first 24 hr of the simulations, especially in the x‐coordinate. During this time, there is a negative x‐offset of the $q_{r}$ field with respect to the $q_{l}$ field in the FS and NS cases of up to 100 m (Figure 11a). A negative offset here means downwind. In the BS case, however, the x‐offset is much weaker and of inconsistent sign. Thus, in the FS and NS cases, rain falls downwind of clouds, while in the BS case, precipitation is located under clouds. Shear tilts clouds (resulting in a higher projected cloud cover, see Figure 7f), which causes part of the rain to fall out of the sides of the clouds: downwind under FS and upwind under BS (as visible in Figure 9). On the second day, the convection becomes more clustered and less random and the offset signal thus more inconsistent. The y‐offset is more incoherent (Figure 11b), suggesting a more random distribution of rain in the meridional direction, but this is not surprising given that the mean wind is in the zonal direction.

The tendency of new updrafts to emerge upwind of existing clouds in the FS and NS cases and then tilt forward (see Figure 9) is because the subcloud layer is characterized by zonal FS (Figure 8g). This means that clouds move faster than their roots (subcloud‐layer thermals), which literally stay behind and can continue to feed moisture into the cloud layer right behind (upwind) of earlier cells. In the BS case, there is only little shear in the subcloud layer, and the wind speed is similar near the ground and at cloud base. This implies that the roots of thermals move at the same speed as the clouds above, making them more vulnerable to precipitative downdrafts, inhibiting the updraft.

## 5 Discussion and Conclusion

**Figure 12:**
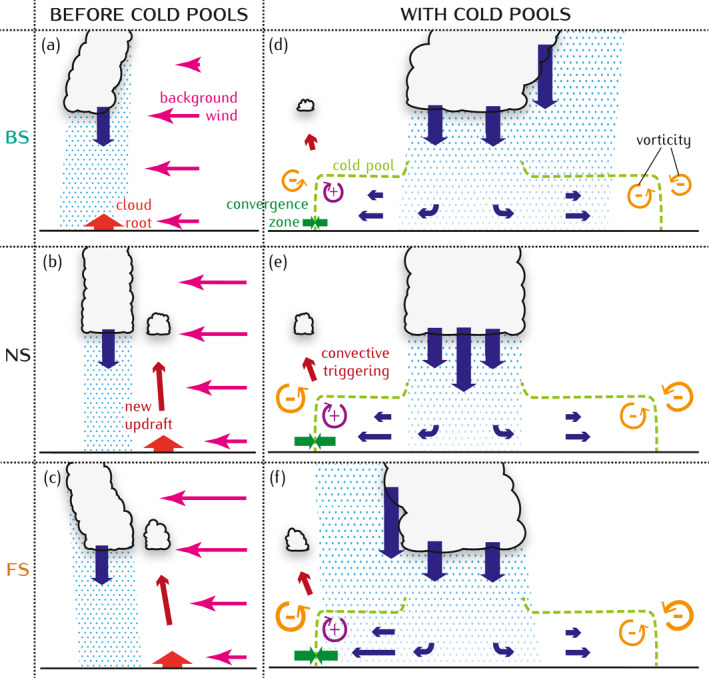
Conceptual picture of (a and b) the morphology of unorganized clouds and (c and d) the structure of cold pools in (a and c) the backward shear case on the one hand and (b and d) the forward shear and no shear cases on the other hand.

In this paper, we used idealized LES experiments with and without cold pools and with different amounts of vertical wind shear, to investigate differences in cloud morphology and the structure of cold pools that develop due to wind shear and that may influence convective development and deepening. We find that shear has an influence on subcloud‐layer circulations by separating updrafts from downdrafts, by setting the area and location of rain and rain evaporation and thus the moistening of the subcloud layer, and by introducing different wind‐speed anomalies through CMT, which may strengthen circulations (divergence and convergence) and convective triggering. We summarize our findings in the schematic in Figure 12:

1. In the BS case, precipitative downdrafts are located near or upwind of existing clouds, which is also where new updrafts are located before cold pools are present (Figure 12a). The precipitation hence hampers new and existing convective cells in their development. In the FS and NS cases, precipitative downdrafts are located downwind, separated from the existing root and new updrafts (Figures 12b and 12c).
2. Once cold pools are present, new convection is typically triggered downwind at the gust‐front outflow boundary, where convergence triggers forced uplift (Figures 12d–12f). There is stronger horizontal convergence at the downwind gust front in the FS and NS cases. This facilitates the formation of stronger updrafts in these cases compared to the BS case.
3. In the FS and NS cases, the subcloud‐layer is characterized by pronounced FS, which implies the presence of negative vorticity, which leads the updrafts to tilt more forward, possibly tapping into moister air ahead of the cold pool (Figures 12e and 12f).
4. Stronger wind‐speed anomalies develop under FS and NS compared to BS, even before cold pools develop and in the complete absence of cold pools. This suggests that CMT facilitates the development of stronger subcloud‐layer circulations by introducing stronger winds and thus stronger divergence in the (raining) downdraft area downwind of existing cells, while introducing relatively weaker winds and thus more convergence in the updraft regions.

The mechanisms in the FS and NS cases are overall similar as indicated in Figure 12, because both cases have subcloud‐layer FS. However, there are still some differences between them. For example, the FS case has a tendency to develop stronger column‐moisture aggregations and deeper clouds at an earlier point in the simulation because this case has larger wind‐speed anomalies and stronger updrafts, indicative of stronger circulations. Furthermore, the FS case has a moister subcloud layer, because of more rain evaporation. Preliminary analysis of simulations run on an even larger domain (150×150 ${km}^{2}$) support our findings here. On this large domain, the FS case develops deep convection with tops >10 km and a large number of cold pools within half a day, while the BS clouds only reach 10 km after more than 40 hr.

After a longer simulation time, the FS case loses its advantage over the NS case as cold‐pool fractions and CTHs are lower. As shown in HSRN2020, this can be attributed to weaker cloud updrafts under FS (and BS) as compared to NS, due to a slanting of the updraft and a stronger downward‐oriented pressure gradient force. Additionally, precipitative downdrafts get weaker under FS, because they are spread out over a larger area due to shear (Figure 12f). Cold pools in the NS case become more vigorous in this stage because precipitation remains concentrated in narrow rain shafts. This is reflected by the significant increase of the variance of $q_{r}$ (while $q_{r}$ itself only increases slightly) from the NS‐NCP to the NS‐STD case (Figures 8a and 8f), that is, when convection transforms from more random organization with precipitation throughout the domain (low variance) to cold pools with narrow strong rain shafts and dry areas surrounding them (high variance). On the other hand, cold pools in the FS case are less vigorous because precipitation is spread out over larger areas, as reflected in the similar variance of $q_{r}$ in the FS‐STD and FS‐NCP cases (Figure 8f). Furthermore, rain falling at the same downwind location where cold pools trigger, new convection (see Figure 4a) inhibits the FS case. The disadvantage of the BS case is diminished by the relocation of convective triggering to locations upwind instead of downwind once strong precipitative downdrafts lead to the formation of cold pools.

Overall, the cloud morphology is thus most favorable for convective deepening if FS is present in the subcloud layer (FS and NS cases) but no FS in the cloud layer (NS and BS cases). In the BS case, the low amount of shear in the subcloud layer and the presence of shear in the cloud layer is disadvantageous for cloud deepening, while in the FS case, only the cloud‐layer shear forms a disadvantage. The NS case can ultimately develop the deepest clouds and most cold pools because it combines all advantages: FS in the subcloud layer and a lack of shear in the cloud layer.

HNRS20 showed that simulations with interactive surface fluxes have a similar response to wind shear as those with constant surface fluxes, and preliminary analysis suggests that this is also the case for the cold‐pool characteristics presented here. Furthermore, Gentine et al. (2016) suggest that interactive surface fluxes are only of importance for cold pools over land and much larger cold pools, but further work on this question is ongoing (e.g., in the framework of ${EUREC}^{4}$A; Stevens et al., 2021). It should be noted that a thermodynamic mechanism involving surface‐flux feedbacks may also trigger secondary convection (Tompkins, 2001), but surface enthalpy fluxes were prescribed in our simulations. Because surface‐flux feedbacks are absent, and the only difference between the simulations is the wind shear, our study provides evidence that proposed mechanisms of triggering secondary convection through moisture convergence at cold‐pool edges (e.g., Böing et al., 2012; Mulholland et al., 2021; Schlemmer & Hohenegger, 2014) and through mechanical uplift (e.g., Li et al., 2014; Meyer & Haerter, 2020) may be facilitated through CMT, which is known to matter for deep convective organization. This underlines the notion that it is not a single mechanism that is responsible for the triggering of secondary convection at cold‐pool gust fronts (Torri et al., 2015).

## Data Availability

The exact version of the code as well as the input files used in this work are available at https://doi.org/10.5281/zenodo.4668479.
